# DataPype: A Fully Automated Unified Software Platform for Computer-Aided Drug Design

**DOI:** 10.1021/acsomega.3c05207

**Published:** 2023-10-12

**Authors:** Mohemmed Faraz Khan, Shubhangi Kandwal, Darren Fayne

**Affiliations:** § Molecular Design Group, School of Biochemistry and Immunology, Trinity Biomedical Sciences Institute, Trinity College Dublin, Dublin 2, Ireland; ‡ Department of Pharmaceutical Chemistry, Faculty of Pharmacy, Integral University, Lucknow U.P., 226026, India

## Abstract

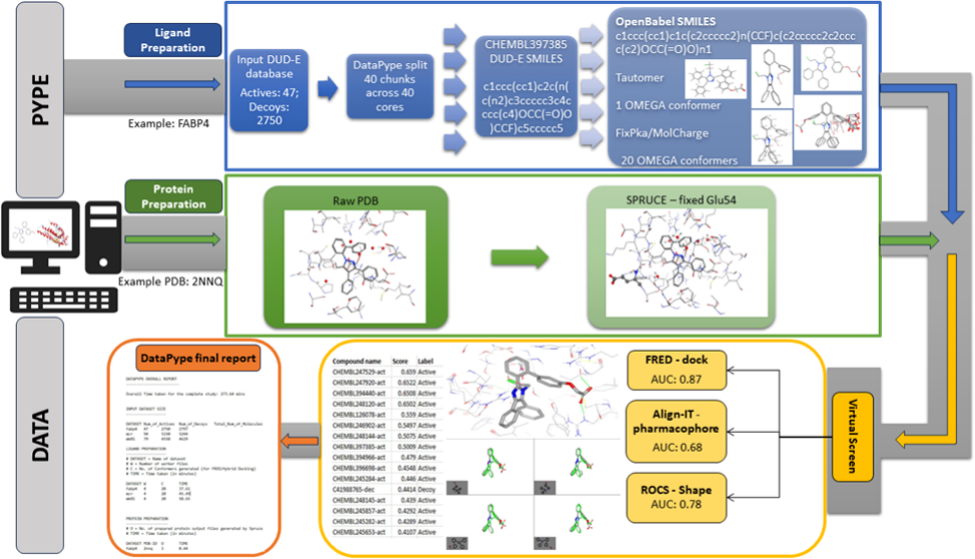

With the advent of computer-aided drug design (CADD), traditional physical testing of thousands of molecules has now been replaced by target-focused drug discovery, where potentially bioactive molecules are predicted by computer software before their physical synthesis. However, despite being a significant breakthrough, CADD still faces various limitations and challenges. The increasing availability of data on small molecules has created a need to streamline the sourcing of data from different databases and automate the processing and cleaning of data into a form that can be used by multiple CADD software applications. Several standalone software packages are available to aid the drug designer, each with its own specific application, requiring specialized knowledge and expertise for optimal use. These applications require their own input and output files, making it a challenge for nonexpert users or multidisciplinary discovery teams. Here, we have developed a new software platform called DataPype, which wraps around these different software packages. It provides a unified automated workflow to search for hit compounds using specialist software. Additionally, multiple virtual screening packages can be used in the one workflow, and if different ways of looking at potential hit compounds all predict the same set of molecules, we have higher confidence that we should make or purchase and test the molecules. Importantly, DataPype can run on computer servers, speeding up the virtual screening for new compounds. Combining access to multiple CADD tools within one interface will enhance the early stage of drug discovery, increase usability, and enable the use of parallel computing.

## 1 Introduction

The number of small molecules that can be purchased for a biological assay against a disease-relevant target has recently exceeded 6 billion (Enamine REAL; https://enamine.net/compound-collections/real-compounds/real-database), traversing well beyond the historically happy hunting grounds of Lipinski-like chemical space.^1^ Publicly available databases such as ChEMBL^2,3^ contain biological assay data for ∼2.3 million compounds, a tiny fraction of chemical space, but these databases are indispensable in data-driven computer-aided drug design (CADD) applications.^4^

The traditional approach for the discovery and development of novel drugs to treat human disease involving physically making and biologically testing thousands of molecules before clinical trials on humans has been superseded by a more targeted approach. The rise of target-focused drug discovery has developed concurrently with the uptake of computer-aided drug design (CADD) across the pharma, biotech, and academic research communities. CADD has also benefitted enormously from access to hardware advances (storage, CPU, and GPU) and algorithm developments. CADD software can now be used to predict molecules that are more likely to modulate the activity of a protein involved in disease pathogenesis prior to making them, focusing more quickly on molecules that have the potential to become drugs.^5,6^

Based on the availability of a 3D protein structure and prior knowledge of target protein biological function(s), CADD is divided into two main approaches: structure-based drug design (SBDD) and ligand-based drug design (LBDD). SBDD approaches utilize protein 3D structures to design compounds that are anticipated to bind with high affinity to the target protein.^7^ In the absence of a protein 3D structure, LBDD is a widely used approach. The knowledge of known active compounds’ chemical and structural characteristics required for binding to the target is utilized in LBDD approaches.^8^

Typical LBDD methods include property-based filtering libraries of compounds,^1^ pseudoreceptor modeling,^9^ pharmacophore searching and filtering,^10−12^ and shape matching.^13,9^ Structure-based drug design (SBDD)^14^ methodologies include molecular dynamics,^15^ docking/scoring, and analysis,^16^ all by a variety of software packages. Artificial intelligence techniques are leveraging the increasing availability of data and computational resources to have a positive impact across all aspects of CADD.^17,18^ For a comprehensive review of computational drug design, see Sliwoski et al.^19^ Each of these standalone applications have their own input and output files and expert knowledge requirements to extract optimal results and some may only run on a single CPU core. For those that can run calculations over multiple cores, different underlying protocols are often used; for example, OpenEye uses OpenMPI, while MOE uses a scalable multiprocessor (SMP) approach to run virtual screening (VS) on multiple cores. Thus, the user is required to have in-depth knowledge of file formats, conversion tools, and optimal parallel computing parameters for each software tool. For the nonexpert user or for those involved in multidisciplinary discovery teams, this can be a daunting challenge.^20^ With virtual collections of molecules ever expanding (over 37 billion commercially available compounds on the ZINC-22 website^21^), turn-around time is key in VS experiments and necessitates parallel computing approaches.

Chemical and biological data used to train CADD software are heterogeneous, often internally inconsistent, and require significant cleaning.^22^ Each CADD tool requires different data preprocessed in a particular way and “user expertise” to optimize how the tool learns from the data. Inherent in the setup is the inability to reproducibly apply the approach to another protein target due to the manual nature of the optimization process. This inhibits the sharing of “best practise” (https://cache-challenge.org/^23^) with team members and necessitates reinventing the whole process again for a new project. Hence, there is a significant need to streamline the sourcing of data from different databases and automate the processing/cleaning of data into a form usable by multiple CADD software packages. The correct curation of molecule/protein data will give higher quality data sets for CADD studies.^24,25^ DataPype does not explicitly take into account the physiological context of the VS campaign, such as the cellular or organ location of the protein target, so consideration of the protonation state of the screening database compounds at different pHs requires manual editing of the associated command line by the user. Seeking to discover covalent ligands or working on a binding pocket located at a PPI would also be not well suited to study with this technology.

Various techniques provided by hundreds of software packages are available to aid the molecular designer in the search for bioactive molecules; each one of them is useful in a particular confined area of drug design.^26^ Many of these are freely available for academic use but usually do not come with the support, ease of installation, user-friendly GUI, and documentation of commercial packages. Typically, CADD software tools are applied singly or in a haphazard manner to discover and develop novel small molecules to bind to and modulate the activity of a protein involved in disease progression. Running VS campaigns for early phase drug discovery on high-performance computing systems usually involves learning to develop software or using existing technologies to run calculations. There is a steep learning curve associated with using each technique proficiently, optimizing the software for a project, and maximizing the utility of parallel computing. Specialist knowledge and expertise is needed to get the best out of each technique and package to prepare the input data and analyze the output—the software needs to know the desired physical and chemical properties of a “drug” so that it can learn to predict possible new drugs.^27^

The availability and capability of HPC infrastructure are important aspects that can determine the viability of a VS campaign. Many academic research groups have a local server for running smaller jobs and initial benchmarking, in addition to access to a university managed HPC system and often links to the national HPC infrastructure. Each system is unique, requires familiarity with different queueing systems, such as SLURM, and offers varying levels of resources. Run allocations are often time-restricted on compute nodes, so any serial calculations can cause a breach of these limits. Multiple commercial offerings are available to compete with local HPC resources such as Google Cloud Computing, Azure, and AWS. These offer automated machine learning services in addition to pay-as-you-go compute and storage. Academic groups can have difficulties budgeting for and absorbing such costs. Commercial CADD software developers are also offering their customers online solutions to their computing needs such as OpenEye Orion^28^ and an equivalent offering from Schrödinger.^29^ These services offer limited interoperability with other systems and are often linked to predetermined HPC offerings.

An increasingly problematic issue in developing CADD software processes is the complicated ecosystem of chemo- and bioinformatics libraries that are often required to build each application. Both the initial installation, in addition to subsequent update and maintenance operations, often generate compatibility issues, redundancies, and ambiguities. Proper use of these libraries requires a high level of user expertise.

To circumvent these issues, CADD platforms have been developed that combine the functionality of multiple standalone algorithms. Many aspects of CADD have been incorporated within various platforms, such as generator user interface (GenUI), which enables the integration of de novo molecular generation techniques^30^ for data preprocessing, model building, molecule generation, and interactive chemical space visualization, within a GUI and also provides APIs to integrate with other tools. The molecule generator emphasis and the descriptor-focused QSAR modeling of GenUI are major differences to DataPype.

Another LBDD-focused platform is LigAdvisor which is a web server enabling the combined application of a number of ligand-based similarity approaches.^31^ A locally installable version is not available. SBDD approaches have also been combined within platforms such docking in Octopus^32^ and MD & quantum mechanics within VIKING.^33^ The ezCADD platform incorporates most of the commonly used CADD techniques within one platform but is only available online.^34^ Another powerful open-source data analytics and integration platform is KNIME (Konstanz Information Miner), allowing the user to create flexible workflows to integrate data from various sources, perform data preprocessing, run predictive modeling algorithms, and visualize results.^35^

We have shown previously that the use of two or three complementary CADD tools yields higher quality hit molecules.^16,36^ Compounds highly ranked by multiple CADD approaches should be more likely to be active.^37^ Multiple tools are available for sequential use within the described data pipeline. With the overall aim of streamlining the early stage drug discovery process, we have created a homogeneous fully automated parallel dataflow software platform for performing small-molecule hit finding and hit-to-lead development, called “DataPype”. It wraps around existing CADD tools to create a single interface to distribute calculations over multiple CPU cores. DataPype can apply a combination of multiple open- and closed-source CADD approaches—docking (FRED^38^), shape (ROCS^39^), and pharmacophore (Align-it)^40^ in parallel against a protein target to identify hit compounds and combine the results. If different levels of abstraction for modeling potential hit compounds all predict the same set of molecules, we have higher confidence that we should synthesize/purchase and test them. Importantly, DataPype can run on computer servers, therefore speeding up searches for new compounds and making use of the Big Data that are feeding into pharmaceutical drug discovery and development.

## 2 Computational Methods

### 2.1 General Overview of DataPype

DataPype is coded in Python 3.5, within the Ubuntu 20.04 OS running in WSL2 on a Windows 10 Education edition, 22H2 PC, to wrap around different CADD tools, split molecule databases into smaller chunks, pass data (molecules and proteins) in parallel through the different steps of the CADD protocol, and combine all processed chunks at the end for analysis and reporting. It is underpinned by several cheminformatics software such as OpenBabel^41^ and RDKit^42^ and data science libraries including NumPy,^43^ Pandas,^44^ scikit-learn,^45^ and SciPy.^46^ It currently includes scripts to pass data to OpenEye (OMEGA,^47,48^ Tautomers,^49^ FixpKa,^49^ and MolCharge^49^), OpenBabel, and RDKit for ligand preparation, whereas protein preparation is supported by OpenEye (SPRUCE), PDBFixer,^50^ and LePro.^51^ DataPype can employ different CADD VS approaches and software including docking (FRED), shape (ROCS), and pharmacophore (Align-it).

DataPype is installed as a new Conda environment using a yml file. OpenEye, Align-it, and other applications are installed separately. After installation, a folder called DataPype is created that contains all of the python working scripts and a text-based configuration.ini file. This configuration file specifies the main DataPype settings such as the protein target ID, number of database chunks, if the ligands should be prepared, the number of conformers to generate, to use all or a subset of the docking, shape, and pharmacophore software.

There are two approaches through which DataPype functions: the benchmarking mode and the virtual screening mode. The benchmarking mode is designed to support the verification of the efficiency of CADD software utilizing benchmarking data sets such as the Database of Useful Decoys-Enhanced (DUD-E)^52^ or DUD-E+^53^ through analysis of metrics calculations. It is important to note that the benchmarking data sets, like DUD-E and DUD-E+, consist of a finite set of targets and screening ligands. Consequently, the results generated in the benchmarking mode should ideally be identical for all users of DataPype and other CADD software, assuming consistent configurations and parameter settings. Therefore, the benchmarking mode not only serves as a tool for evaluating software installation but also contributes to the standardization and validation of computational workflows in the field of drug design. VS mode screens chemical databases to discover potentially therapeutically active small molecules. The mode for running calculations, input molecules and target files, and the required parameter settings, in addition to a short explanation of each, are specified in the configuration .ini file. Once these parameters are defined in the working directory, DataPype starts calculations by running the command: python Master_DataPype.py Config_DataPype.ini.

Master_DataPype.py is a master script that calls other scripts to perform different tasks specified in the configuration file. In the VS mode, DataPype can fetch activity and compound data of bioactive molecules from the ChEMBL database. It can also download the associated protein data from the RCSB PDB database.^54^ In benchmarking mode, DUD-E data sets are available and are utilized to perform benchmarking of different CADD approaches by calculating and comparing different benchmarking metrics.

DataPype performs CADD calculations in a fully automated fashion, therefore reducing the time and effort required in comparison to manual performance. The input database of small molecules is divided into a number of chunks (equal to the number of cores to be used) for parallel calculations locally or on a server using Master/Slave paradigms. As a platform composed of multiple tools and software, DataPype supports parallel calculations on CPU cores. The utilization of GPU acceleration depends on the available hardware and the capability of each software application included within DataPype. For example, OMEGA calculations can be chunked over multiple CPU cores, and if required, each chunk can benefit from torsion driving on a GPU. Users should consult relevant software documentation for guidance on GPU integration. Another significant feature of DataPype is that it is scripted to streamline the input data and output results in a clean and organized manner with systematic segregation of directories. As determined by the user, the output files from one directory can be automatically selected and used as input files in subsequent calculations. At the end of each step, a concise report is generated, detailing key results from each step, i.e., listing molecules failing/passing the step. All of the generated reports are compiled into one master report at the end of each study performed by DataPype. A sample report is provided in the Supporting Information.

### 2.2 Sourcing of Data

The benchmarking mode of DataPype is designed to take input data from DUD-E. DUD-E contains data sets of experimentally verified actives and property-matched decoys for a total of 102 targets, with an average of 224 active ligands per protein target and 50 decoys per active, along with a crystal structure of each target.

In the VS mode, DataPype has the option of fetching activity and compound data of bioactive molecules from the ChEMBL database using an automated python script. Cleaning and processing of the data are also performed by the script. DataPype can also download protein data from the RCSB PDB database using a script with the PDB REST-based API that takes the UniProt protein ID code as input and searches for corresponding X-ray structures of this target in the PDB, collates and filters structures based on certain criteria, such as resolution, confirms that a ligand is present, and downloads the metadata and structures of the proteins and cocrystallized ligands with the lowest resolutions.

### 2.3 Processing of Molecules and Protein Data

#### 2.3.1 Ligand Processing

Ligand processing in DataPype comprises ligand preparation and ligand sanitization. In the ligand sanitization step, all of the duplicate molecules were removed, as were salts and dative bonds were fixed. This step was performed by OpenBabel. Ligand preparation includes the generation of tautomers, enumeration of ionization and protonation states, calculating partial charges, and conformer generation. This step was primarily performed with OpenEye’s QUACPAC (Tautomers, FixpKa, MolCharge) and the OMEGA (conformer generation). Comparison was also made with the corresponding functionalities provided by RDKit and OpenBabel.

#### 2.3.2 Protein Processing

DataPype uses OpenEye’s SPRUCE as its default tool to prepare protein structures. After downloading the structure for a given PDB ID from the PDB website, the structure was protonated, missing side chains and loops were built, alternate locations were enumerated, and tautomers for the cocrystallized ligands and cofactors were enumerated and evaluated by SPRUCE. The output files generated from SPRUCE were named according to the default general naming conventions of the software.

### 2.4 CADD Approaches

Multiple software types are supported by DataPype. Within our initial platform, we incorporated fast software from three of the main CADD subdisciplines, structure-based, shape-based, and pharmacophore-based drug design, which are freely available for academic use. For docking, OpenEye’s FRED is used; for Shape, OpenEye’s ROCS is used; and for Pharmacophore, Align-it is used. On annual renewal of the OE academic license, the user is asked for proof of citation of OE tools in recent publications.

### 2.5 Benchmarking Studies

Three rounds of benchmarking were undertaken to stringently test DataPype’s capabilities and performance using the DUD-E data set. DUD-E consists of 102 targets, and each target consists of a preprocessed PDB structure and a set of labeled active and decoy molecules. The actives and decoys were combined into a haystack file, which at the beginning of a DataPype run was subdivided (chunked) into a user-defined number of “worker” files, each running on one CPU core. The majority of the results described in this article are for 40 worker chunks running in parallel on 40 cores. The performance of the CADD approaches was evaluated by using performance metrics including ROC-AUC and EF1%;^55^ further details of these metrics are provided in Section 2.6. The difference in the time taken by various methods was also compared. All large-scale benchmarking calculations were performed on 1 node (consisting of 2 × 20 cores 2.4 GHz Intel Xeon Gold 6148 (Skylake) processors) of the Kay supercomputer at the Irish Centre for High-End Computing (ICHEC).

#### 2.5.1 Optimization of the Individual CADD Preparation Software Parameters

There are several internal parameters in each of the included cheminformatics and CADD tools that require optimization. All such parameters were adjusted and compared to the standard protocol for performing ligand preparation (by OpenEye, OpenBabel, and RDKit) and protein preparation (by SPRUCE, PDBFixer, and LePro). These parameters were subsequently used in docking (FRED), shape (ROCS), and pharmacophore (Align-it) screening. Default values were used for FRED, ROCS, and Align-it. This also directed the choice of the best default tools and their parameter values to be assigned in DataPype.

As there are a large number of options to be examined for many of the parameters, it was not feasible to run studies on the complete DUD-E data set. Therefore, we used a representative subset of DUD-E comprising of targets that only yielded one output file after protein preparation by SPRUCE. Then among these shortlisted data sets, we further selected 7 diverse data sets across different protein families containing the smallest number of molecules across different protein family categories (Table 1).

**Table 1:** Data Sets Selected for Internal Parameter Optimization and Selecting the Default Tools for Performing Ligand Preparation, Protein Preparation and CADD Approaches, Their Protein Family, and the Number of Ligands

data set	category	haystack size
cp2c9	CYP450	6082
adrb2	GPCR	15 225
gria2	ion Channel	11 991
mk01	kinase	4628
fabp4	lipid transport	2796
mcr	nuclear	5241
fa7	protease	6360

#### 2.5.2 Benchmarking of Individual CADD Software Included in DataPype

The software selected as default (FRED, ROCS, and Align-it) for all three approaches (docking, shape and pharmacophore) included in DataPype, using their optimized parameters, performed a full validation run against the DUD-E data sets (all 102 targets). The resulting scores obtained after performing the benchmarking were used to calculate the performance metrics mentioned above and were compared to those of previously published work.

### 2.6 VS Scoring Metrics

Two of the most widely used metrics, AUC and EF1% scores, were used to assess the ranking performance of the VS packages incorporated within DataPype. AUC integrates the receiver operating characteristic (ROC) curve generated by plotting the ranking of the true and false-positive compounds and represents the probability that any active is more highly ranked than any decoy, and a value of 1 represents perfect performance. The second enrichment factor metric, EF1%, corresponds to the ratio of actives versus decoys in the top 1% of the ranked hitlist over the actives versus decoys ratio in the complete data set (haystack).

## 3 Results

### 3.1 Benchmarking Approaches to Chunk the Molecular Database Input File

In order to examine the effect of molecule ordering within the chunked files on computational efficiency and its subsequent effect on VS performance in terms of computational time, we conducted Smina^56^ docking. This study utilized the fabp4 data set from the DUD-E database and was performed against the 2NNQ protein X-ray structure with exhaustiveness 8, autobox add 4, 10 worker files, and 10 cores. Initially, the molecules in the input database file were divided into chunks sequentially, and each chunk was docked in parallel on separate cores. We found that the time taken to complete the calculations of a few chunks containing more complex molecules, with a higher-than-average number of rotatable bonds, was far longer than that of chunks containing molecules with fewer rotatable bonds. As each step in DataPype needs to finish before the next step begins, this slowed the progress of the VS pipeline. Therefore, a new method of chunking files was implemented to sort molecules by the number of rotated bonds and add them in sequence to each chunk, thereby evenly distributing molecules by the number of rotatable bonds. The new method of chunking files resulted in ∼30% faster Smina-distributed calculations compared to sequentially chunking worker files as shown in Table 2.

**Table 2:** Benchmarking of the Time Taken to Perform Smina Docking of the fabp4 Data Set Using Different Chunking Methods

chunking data set method	time (h)
sequential	10.10
number of rotatable bonds	7.23

### 3.2 Optimization and Benchmarking of the Ligand Preparation Method

**Figure 1:**
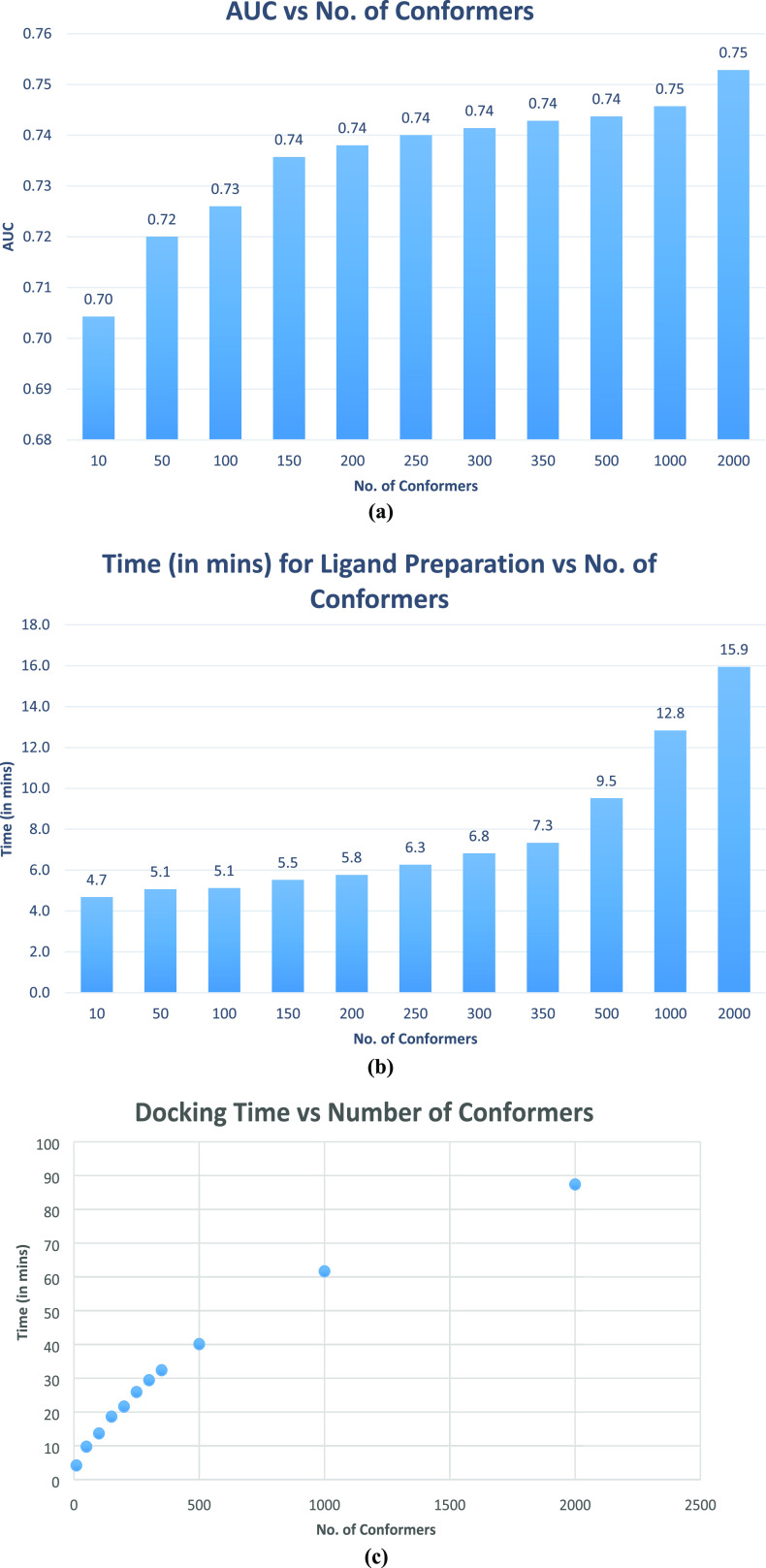
Graphs representing the average of (a) ROC-AUC values, (b) time taken for ligand preparation, and (c) time taken (minutes) for performing FRED docking with different numbers of conformers of the seven target DUD-E subsets.

A protocol to perform ligand sanitization and preparation was designed using OpenEye in conjunction with OpenBabel, an open-source tool, on 40 chunks/cores. This involved (a) canonicalizing SMILES and fixing dative bonds with OpenBabel, (b) tautomer generation (QUACPAC Tautomers), (c) enumerating of ionization states and protonation (QUACPAC FixpKa), (d) calculating partial charges (QUACPAC MolCharge), (e) conformer generation (by OMEGA), and (f) a final deduplication step in the case of formation of duplicate tautomers or protomers with OpenBabel. The inclusion of OpenBabel alongside OpenEye was driven by our commitment to maximize the utilization of open-source solutions while providing flexibility for users. Each software package has parameters for optimization such as the number of tautomers and conformers to be generated and which force field to be used for partial charges. To study the impact of changing these parameters on the performance of DataPype, different ligand sets were prepared by changing one parameter at a time, keeping other values fixed, and examining the effect on the ROC-AUC and EF1% values after performing FRED docking with these prepared ligands. This study was performed on the seven DUD-E data sets described in Section 2.5.1, and the average results were calculated. If the result was better than the default, the change was accepted and added into the new default protocol; otherwise, no change was made to the default protocol. The results of this study are shown in Table 3 and Figure 1a–c. For all results, the AUC values are shown to two decimal places and EF1% and time to one decimal place.

**Table 3:** Benchmarking and Optimization of the Ligand Preparation Methodology Based on Benchmarking Metrics (AUC and EF1%) Calculation and Time (Minutes) Required to Complete Ligand Preparation and Docking (Using FRED)^a^

	average benchmarking metrics values			
method/parameter	AUC	EF1%	time (docking)	time (ligand preparation)
default_DataPype	0.72	18	9.7	5.1
optimization of variable parameters of different steps of openEye				
1. Tautomers				
(i) stereo_LocalSampled	0.72	18.2	9.7	5.1
(ii) stereo_EverSampled	0.72	18.2	10.1	6.1
(iii) taut_maxgenerat_4096 (default value of openEye)	0.72	18	9.7	5.1
(iv) CH3_False	0.72	18	9.7	5.7
(v) PKA_False	0.72	18.1	9.6	5.3
(vi) maxTautomericAtoms_140	0.72	18	9.7	5
(vii) taut_MaxZoneSize_70	0.72	18.1	9.4	5.2
2. Conformers				
(i) 10	0.7	13.8	4.1	4.7
(ii) 50 (default)	0.72	18	9.7	5.1
(iii) 100	0.73	18.3	13.7	5.1
(v) 200	0.74	19.3	21.6	5.8
(vi) 250	0.74	19.3	25.9	6.3
(vii) 300	0.74	19	29.4	6.8
(viii) 350	0.74	19.6	32.4	7.3
(ix) 500	0.74	19.5	40.1	9.5
(x) 1000	0.75	20.5	61.7	12.8
(xi) 2000	0.75	21.2	87.4	15.9
3. MolCharge				
(i) am1bccsym	0.72	18	9.4	27.7
(ii) mmff (default)	0.72	18	9.7	5.1

a The values represented here are the average of the results obtained for the seven selected DUD-E data sets ran in triplicate.

Our analysis showed that the default parameters used for the OpenEye ligand preparation software (Tautomers and MolCharge) were appropriate as they were. We decided to generate 200 conformations in future studies, as this gave the best AUC versus time balance. After optimizing and establishing the best parameter values for the OpenEye-based protocol, we replaced each step with the RDKit or OpenBabel equivalent and determined the impact on the benchmarking metrics to see if either was a better alternative. Two additional protocols were designed using only the RDKit or OpenBabel functionality. The comparison of these results with the benchmarking metrics results of the OpenEye method is detailed in Table 4.

**Table 4:** Benchmarking of the Ligand Preparation Methods by RDKit and OpenBabel in Comparison to the OpenEye Methods Based on Benchmarking Metrics (AUC and EF1%) Calculation and Time (min) Required to Complete Ligand Preparation and Docking (Using FRED)^a^

	average benchmarking metrics values			
	AUC	EF1%	time (docking)	time (ligand preparation)
new default dataPype	0.74	19.3	21.6	10.6
optimization by changing different steps with openBabel or RDKit				
1. OpenBabel				
(i) protonate at pH 7.4 by openBabel, other steps with OE	0.73	17.2	19.3	5.0
(ii) partial charge (Gasteiger) by openBabel, other steps with OE	0.72	18.5	22.8	5.8
(iii) partial charge (mmff94) by openBabel, other steps with OE	0.72	18.5	22.9	5.8
(iv) 200 conf generation by openBabel, other steps with OE	0.72	18.2	34.9	349.9
complete ligand preparation (all steps) by openBabel	0.72	17.9	31.9	305.9
2. RDKit				
(i) tautomer by RDKit, other steps with OE	0.74	19	20.0	5.0
(ii) 200 conf generation by RDKit, other steps with OE	0.7	12.5	36.0	260.3
complete ligand preparation (all steps) by RDKit	0.67	8.7	27.9	260

a 200 conformers were generated with OMEGA except where stated otherwise.

As shown by the docking metrics, the ligand protonation/tautomer creation steps and assigned molecular charges were not improved by using RDKit or OpenBabel; in particular, the conformer generation steps took significantly longer than with OMEGA.

### 3.3 Optimization and Benchmarking of the Protein Preparation Method

The performance of different open-source methods for preparing proteins, OpenEye’s SPRUCE, PDBFixer, and LePro, were compared. Docking using FRED was performed for the seven DUD-E data sets using the proteins prepared by the three software packages, and their metrics were calculated and compared. The results are shown in Table 5.

**Table 5:** ROC-AUC and EF1% Comparison for Docking Performed Using Proteins Prepared by PDBFixer, LePro, and SPRUCE Toolkits

	ROC-AUC			EF1%		
data set	PDBFixer	LePro	SPRUCE	PDBFixer	LePro	SPRUCE
adrb2	0.61	0.61	0.78	6.4	6.4	7.8
cp2c9	0.67	0.67	0.6	2.3	2.3	6.4
fa7	0.74	0.75	0.78	39.7	39.7	43.4
fabp4	0.87	0.75	0.87	21	31.8	22.96
gria2	0.67	0.67	0.72	20.8	19	23.9
mcr	0.69	0.69	0.6	16.5	16.5	15
mk01	0.63	0.63	0.82	13.5	13.5	13.9
average	0.69	0.68	0.74	17.2	18.5	19

Our studies showed that SPRUCE was the best performing protein preparation tool, as judged by subsequent FRED docking performance. The default ligand preparation steps were followed, and 200 conformers were created for each ligand. For each of the seven targets taken from the DUD-E data set, SPRUCE was equivalent to or better than PBDFixer and LePro. However, running a two-tailed *t*-test found that SPRUCE was only better than PDBFixer when considering EF1% values (*P* < 0.05).

### 3.4 Benchmarking of Individual CADD Software Included in DataPype and Comparison with Published Data

After optimizing the different CADD preparation approaches within DataPype (optimized parameters are provided in the Supporting Information), a full benchmarking study was performed using these CADD approaches (FRED, ROCS, and Align-it) against the complete 102 DUD-E data sets. The optimized ligand and protein preparation steps were also used. The benchmarking metrics are detailed in Table 6.

**Table 6:** ROC-AUC and EF1% Values for Different CADD Approaches within DataPype against 102 DUD-E Data Sets

	ROC-AUC			EF1%		
data set	FRED	ROCS	align-it	FRED	ROCS	align-it
aa2ar	0.64	**0.72**	0.57	3.1	**24.4**	1.5
abl1	**0.74**	0.58	0.69	15.9	**17**	11
ace	**0.77**	0.74	0.71	**17.6**	12.5	8.6
aces	**0.74**	0.47	0.52	**13.7**	3.4	5.7
ada	0.73	**0.92**	0.88	17	**52**	34
ada17	**0.78**	0.72	0.65	**22.2**	11.8	13.4
adrb1	**0.76**	0.53	0.63	6.5	12.2	**12.6**
adrb2	**0.78**	0.51	0.52	7.8	**10**	9.6
akt1	0.60	0.34	**0.65**	2	4.1	**5.8**
akt2	0.64	0.41	**0.64**	5.1	10.2	**15.3**
aldr	0.68	0.66	0.61	18.1	34.4	24.4
ampc	0.72	0.74	0.73	32.5	31.1	27
andr	0.68	0.71	0.61	17.3	15.6	14.1
aofb	0.72	0.40	0.48	1.6	3.3	3.3
bace1	0.55	0.32	0.64	15.5	1.7	4.1
braf	0.73	0.56	0.61	20.3	28.2	24.9
cah2	0.63	0.59	0.75	4.6	5.2	4.7
casp3	0.53	0.48	0.53	3.5	1.5	2
cdk2	0.75	0.70	0.63	11.8	14.7	9.9
comt	1.00	0.88	0.93	94.2	36.2	36.3
cp2c9	0.65	0.46	0.47	8.3	1.7	3.3
cp3a4	0.65	0.52	0.47	4.7	3.9	3
csf1r	0.72	0.68	0.65	26	27.6	25.2
cxcr4	0.89	0.91	0.70	30.6	41.9	33.6
def	0.90	0.84	0.93	32.5	40.9	47.2
dhi1	0.78	0.68	0.56	10.9	14.4	1.8
dpp4	0.76	0.71	0.65	11.1	14.5	6
drd3	0.83	0.55	0.52	13.6	5	2.7
dyr	0.61	0.74	0.73	12.7	35.5	23.4
egfr	0.67	0.78	0.78	3.1	19.8	23.3
esr1	0.89	0.77	0.85	43.7	46.2	37.9
esr2	0.92	0.82	0.86	41.3	39	30.6
fa10	0.76	0.71	0.55	17.8	16	8.2
fa7	0.79	0.71	0.58	41.7	21.7	13.9
fabp4	0.87	0.78	0.65	25.1	33.9	34
fak1	0.91	0.94	0.73	47.5	49.5	22.7
fgfr1	0.67	0.52	0.64	5.5	1.4	2.9
fkb1a	0.66	0.59	0.68	6.3	6.4	8.2
fnta	0.70	0.76	0.51	4.3	19.7	2.6
fpps	0.98	1.00	0.97	84.9	88.7	30.8
gcr	0.63	0.54	0.53	15.1	11.2	4.3
glcm	0.64	0.60	0.63	7.3	11	5.5
gria2	0.69	0.70	0.65	16.4	30	11.3
grik1	0.93	0.72	0.68	28.4	11.8	10.8
hdac2	0.89	0.46	0.47	18.7	5.3	5.9
hdac8	0.92	0.76	0.81	29.2	25.9	20.1
Hivint	0.70	0.44	0.55	14.3	2	4
Hivpr	0.69	0.68	0.68	5	6.1	7.1
Hivrt	0.78	0.62	0.60	17.7	12.7	9.3
Hmdh	0.88	0.88	0.89	34.3	51.2	37.3
hs90a	0.88	0.86	0.84	9.6	45.7	13.4
hxk4	0.45	0.82	0.88	4.3	37.6	30.1
igf1r	0.62	0.52	0.67	2	14.1	18.1
Inha	0.89	0.77	0.71	11.3	18.1	15.9
Ital	0.46	0.55	0.59	5.4	13	7.2
jak2	0.86	0.81	0.78	23.1	36.1	22.2
kif11	0.83	0.73	0.60	41.8	20.5	5.1
kit	0.50	0.45	0.28	3	3	1.2
kith	0.87	0.81	0.95	20.4	25.5	50.9
kpcb	0.80	0.77	0.68	36.3	47.6	34.6
lck	0.61	0.59	0.62	15.7	8.2	9.3
lkha4	0.89	0.81	0.81	13.9	31.3	27.2
mapk2	0.77	0.85	0.65	21.9	37.6	19.8
mcr	0.61	0.62	0.61	14.4	13.8	5.3
met	0.82	0.83	0.89	15.6	41.9	59.9
mk01	0.79	0.73	0.74	12.6	26	32.2
mk10	0.76	0.57	0.52	13.4	5.7	5.7
mk14	0.65	0.66	0.53	20.4	14.6	4
mmp13	0.73	0.80	0.75	10.4	25.2	20.3
mp2k1	0.58	0.47	0.44	2.5	14.8	4.9
nos1	0.75	0.42	0.49	8	2	2
nram	0.84	0.90	0.51	11.2	26.5	3.1
pa2ga	0.78	0.76	0.82	23.2	22	4
parp1	0.93	0.77	0.74	38.5	26.3	19.1
pde5a	0.80	0.62	0.70	13.6	26.7	26.7
pgh1	0.68	0.58	0.37	6.1	5.1	2
pgh2	0.73	0.79	0.68	11.4	36.7	21.3
plk1	0.89	0.60	0.56	33.9	1.9	0.9
pnph	0.98	0.94	0.97	56.7	55.7	63.4
ppara	0.82	0.87	0.75	16.9	27.4	15.3
ppard	0.77	0.74	0.51	12.4	12.4	4.2
pparg	0.82	0.80	0.67	18.2	19.7	21.9
prgr	0.81	0.71	0.62	25.7	9.2	0.7
ptn1	0.73	0.38	0.41	25.4	1.5	1.5
pur2	0.83	0.98	0.99	27.3	52.7	54.6
pygm	0.59	0.44	0.43	2.5	1.8	1.7
pyrd	0.87	0.88	0.70	50.1	48.3	47.4
reni	0.51	0.59	0.60	19.4	24.3	16.5
rock1	0.87	0.55	0.45	26.9	1	1
rxra	0.88	0.90	0.28	52.3	30.2	11.4
sahh	0.90	1.00	1.00	42.2	55.5	55.5
src	0.68	0.52	0.57	6.1	2.8	9.2
tgfr1	0.75	0.75	0.84	20.1	17.1	29
thb	0.87	0.89	0.78	39.8	59.5	25.9
thrb	0.70	0.55	0.51	13.3	2.5	1.4
try1	0.73	0.60	0.55	17.8	3.8	1.4
tryb1	0.77	0.51	0.60	15	4.1	5.4
tysy	0.73	0.78	0.81	23.6	19	27.2
urok	0.83	0.56	0.62	45.5	15.4	11.7
vgfr2	0.71	0.61	0.47	9.5	7.8	4.4
wee1	0.99	0.99	0.95	61	61	61.1
xiap	0.90	0.91	0.92	37.9	48.9	42
average	**0.76**	0.68	0.66	20.3	**21.8**	16.8

On average, FRED docking was found to give the best AUC scores, but ROCS yielded the highest EF1% values. Depending on the target though, Align-it can give the best metrics, which highlights the necessity of validating the various CADD tools against the target of interest. Different tools bring an alternative lens to each particular study, which highlights the need for applying multiple parallel CADD approaches.

In order to analyze these results in a broader context, they were also compared with an earlier study by Ericksen et al.^57^ who used multiple CADD approaches on a 21 target subset of the DUD-E data sets; data are shown in Tables 7 and 8. FRED software was used in both studies, but the other software packages were different, although within similar SBDD or LBDD subdisciplines.

**Table 7:**
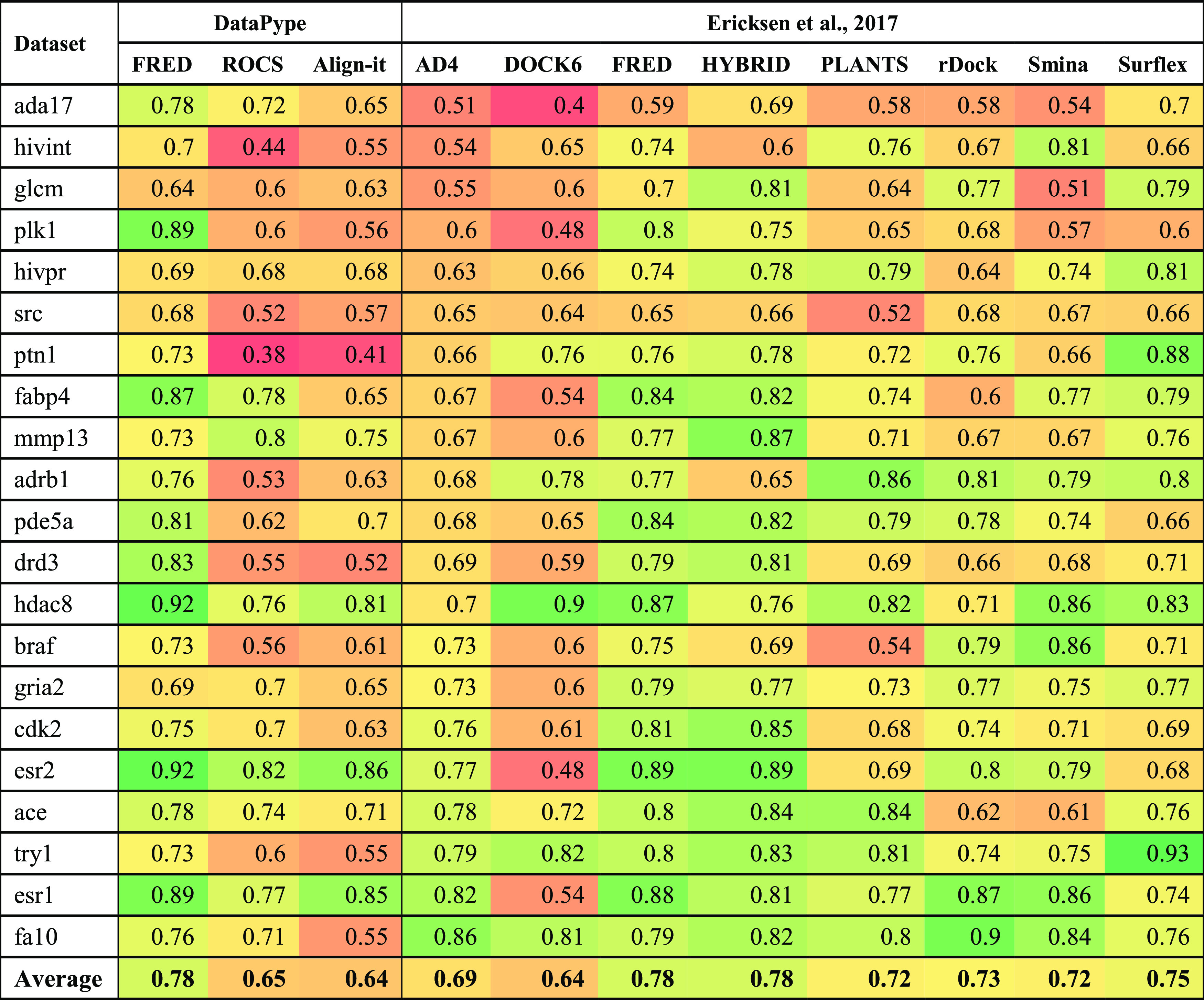
Heatmap of ROC-AUC Values for Different CADD Approaches within DataPype Compared to the 21 DUD-E Data Sets Studied by Ericksen et al.^a^

a For clarity, the best metric scores are in deeper green, while those badly scored targets are progressively in deeper shades of red.

**Table 8:**
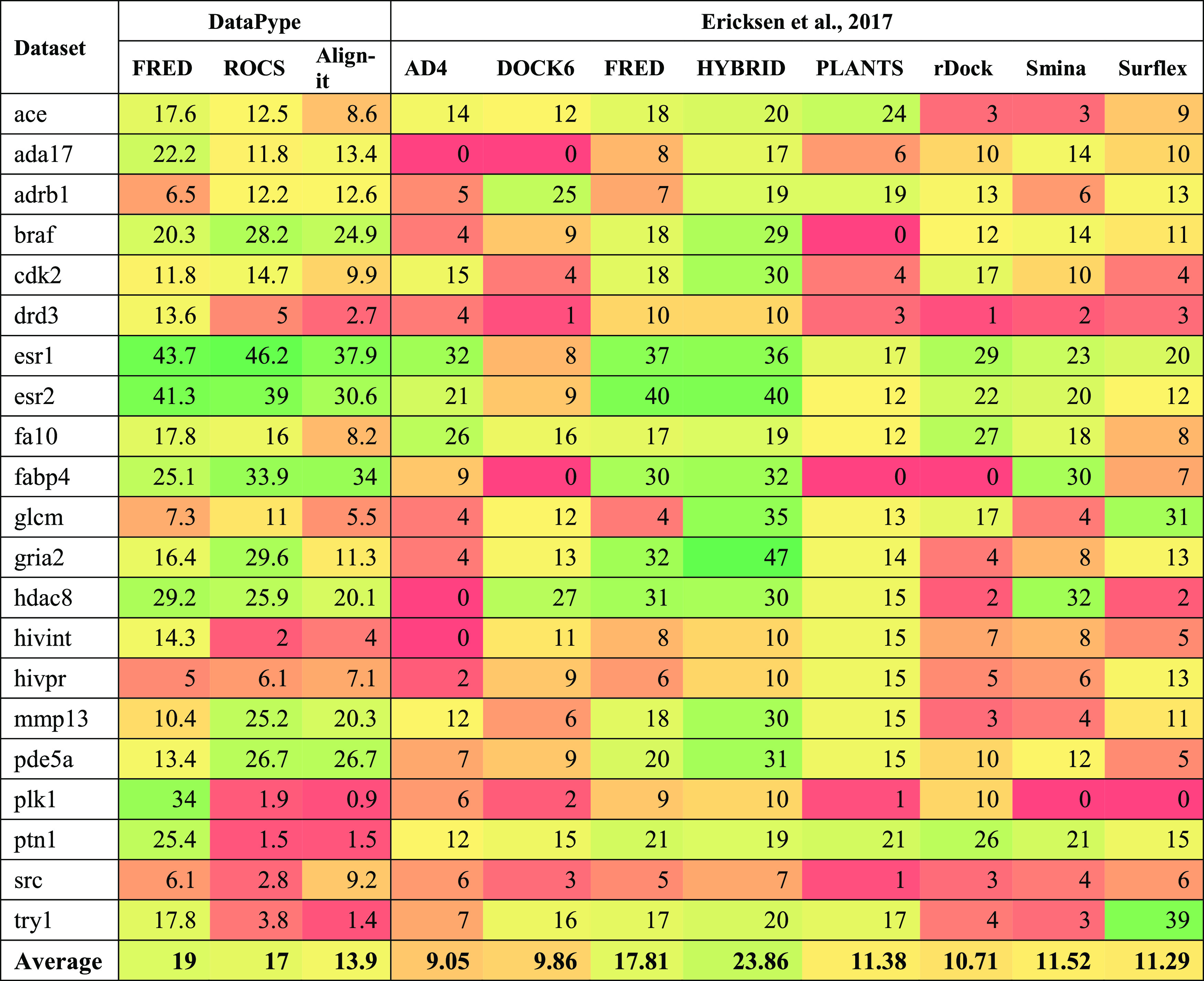
Heatmap of EF1% Values for Different CADD Approaches within DataPype Compared to the 21 DUD-E Data Sets Studied by Ericksen et al.^a^

a For clarity, the best metric scores are in deeper green, while those badly scored targets are progressively in deeper shades of red.

The performance of the three CADD algorithms in DataPype are comparable to or better than the other software studied by Ericksen et al. Some discrepancies were observed between enrichments using FRED in DataPype and those in the Ericksen paper. We have canonicalized and cleaned the target haystack SMILES strings prior to generating 200 conformers of each and used SPRUCE for protein preparation. Ericksen et al. generated 1000 conformations of each ligand directly from DUD-E and used PDB2PQR (v2.1.0)^58^ to process protein structures. This variance in enrichment highlights the importance of every processing step for proteins and ligands. Small changes can have a large impact on VS enrichment. The best consensus scores from the three DataPype tools yielded the same AUC as FRED and HYBRID in the Ericksen paper and performed better than all other software. Running a two-tailed *t*-test on the AUC values confirmed that DataPype consensus was comparable to FRED, HYBRID, and Surflex but better than the other software tools (*P* < 0.05). When considering EF1%, a two-tailed *t*-test confirmed that DataPype consensus was comparable to HYBRID and better than the other software (*P* < 0.05).

## 4 Discussion

The scale of available compounds is now well beyond 6 billion, and software to address and accurately screen such vast chemical spaces is being developed. VirtualFlow is primarily written in Bash and is a large-scale SBDD platform.^59^ It was recently used to VS 1.3 billion compounds against a protein target (KEAP1) using SBDD within a 4-week timespan. Of the 590 hit compounds selected, 69 were confirmed to bind to KEAP1, which is an impressive hit rate. From an LBDD angle, PyRMD is a python-coded platform for applying machine learning techniques within VS campaigns.^60^ They use benchmark and screening modes and can be installed locally but only use one CPU core. Regarding modeling larger-scale molecular processes, such as molecular dynamics, or running studies at a higher level of computational modeling theory, VIKING is an easy-to-use web platform for using standardized workflows to setup virtual studies.^33^ This platform is accessible through a website and offers limited access to different VS technologies.

DataPype seamlessly interlinks different CADD approaches, removing the need for manual user intervention in a VS campaign and enabling all CADD software to benefit from a parallel computing architecture. Widely used tools such as OpenBabel, Tautomers, and MolCharge cannot easily avail of mpi parallel computing, but DataPype has been developed to allow all of these tools to be used across a user-defined number of CPU cores.

One of the most important steps impacting the runtime was the initial splitting of molecules into database chunks for parallel processing. Intuitively, by evenly distributing molecules by their rotatable bond count (increased complexity) over the database chunks, the time spent on each chunk preparing conformers or undertaking tasks that were dependent on molecular flexibility was evened out, resulting in a more balanced runtime. Future iterations of DataPype will also examine the impact of globularity and other structural descriptors on the calculation speed. We will also examine in closer detail the compound database splitting and merging steps to ensure that a throughput bottleneck at these stages does not negatively impact the overall speed of the DataPype protocol.

Our testing of the OpenEye tools’ parameters showed that varying the default values in Tautomers, FixpKa, and MolCharge had very little impact on the docking results of seven DUD-E data sets. Increasing the number of conformers from 10 to 2000 not only steadily increased the docking ranking AUC but also added to the time overhead, both ligand preparation and subsequent docking. We decided on 200 conformers, which was a reasonable compromise between speed and accuracy. For the seven DUD-E data sets examined in this study, using the RDKit or OpenBabel equivalent of the OpenEye calculation did not result in improved docking metrics. Using RDKit to prepare tautomers was equivalent to using OE Tautomers, but none of the other approaches were an improvement. Conformer generation with OpenBabel or RDKit was significantly slower than that with OMEGA.

The importance of using an appropriate protein preparation tool is also highlighted by our study. We examined three freely available applications, and using SPRUCE resulted in a small improvement in docking metrics. LePro adjusts hydrogen atoms and removes water molecules and small molecules. PDBFixer also adjusts hydrogen atoms but in addition builds missing loops. SPRUCE retains water molecules, optimizes hydrogen atom positions, models missing residues and loops, and examines alternate locations. SPRUCE generates individual output files for each cocrystallized ligand, which can be restricted to a single file by specifying the three-letter ligand code of the desired cocrystallized ligand. Additionally, it identifies alternate positions and generates separate output files for each option, but this feature can be disabled by setting the -altloc flag to “primary” instead of “enumerate”. This highlights how essential it is to properly process protein structures prior to utility in docking studies. We plan to evaluate the protein preparation capabilities of commercial software offerings such as MOE and Schrödinger in future releases of DataPype.

In line with our previous studies,^13,16^ our application of DataPype against the whole of DUD-E and comparison with work by Ericksen et al. demonstrated that not one tool performs the best against all targets. The CADD approach taken must be tailored to each target. This is a significant benefit of DataPype as the protocol automates the screening workflow. A few choices such as the target, number of conformers, and CADD tools to be implemented need to be decided, and DataPype, in benchmarking mode, runs the protein and validation haystack molecules through each CADD tool and provides metrics on the most appropriate tool to use. Subsequent VS against a vendor database can then be undertaken by using a subset of the available CADD tools.

## 5 Conclusions

DataPype, a platform for the integration of various VS approaches, splits an input compound database into a user-defined number of chunks for parallel calculations over multiple CPU cores, significantly enhancing the calculation speed. Serial cheminformatic code, such as OE Tautomers and OpenBabel, when wrapped within the DataPype platform, can avail of multicore speed-up, thereby increasing their applicability for processing large compound databases in a reasonable time frame. DataPype is an extendable python framework, so we will incorporate additional open-source and free-for-academic-use applications within the platform and also develop a web-interface for wider dissemination to the research community.

## Data Availability

Unfortunately, our institution does not permit public sharing of code, but we are planning on making the software available on a website soon.

## Supporting Information Available

Optimized parameters for DataPype, platform library dependencies, and an example output report (PDF)
