# Supplementary material for: DataPype: A Fully Automated Unified Software Platform for Computer-Aided Drug Design
Source: ACS Omega. 2023 Oct 12;8(42):39468–80. doi: 10.1021/acsomega.3c05207 (PMC10601415; doi:10.1021/acsomega.3c05207)
Supplement: Supplementary file 1 — ao3c05207_si_001.pdf [file ao3c05207_si_001.pdf]

Supporting Information

DataPype: A Fully Automated Unified Software Platform for Computer-Aided Drug Design

Mohemmed Faraz Khan[a 1], Shubhangi Kandwal[a 1] and Darren Fayne[1]*

[1]Molecular Design Group, School of Biochemistry & Immunology, Trinity Biomedical Sciences Institute, Trinity College Dublin, Dublin 2, Ireland

[a] **Joint first authors**

* **Corresponding Author**

DataPype optimised parameters

Using 7 DUD-E dataset, each parameter was varied and the effect on docking AUC and EF1% was determined.

Reassuringly, overall the default parameters performed very well for all software applications tested.

Ligand preparation

OpenEye Tautomers

The default parameters performed best.

The only flag turned on when running Tautomers was -stereo None

OpenEye fixpka

Used default settings

OpenEye oeomega

Using -strict false we found that 200 conformers gave the best performance versus time ratio.

OpenEye molcharge

The mmff option gave the best performance so -method mmff was used.

Using the equivalent parameters in OpenBabel or RDKit gave no improvement in performance.

Protein preparation

The initial comparison of the three tools found SPRUCE to be the best performing algorithm.

PDBFixer and LePro are not available in DataPype.

The following parameters were used with *SPRUCE*

-altloc enumerate -build_cterm_caps false -build_nterm_caps false

VS CADD software

Default parameters were used for FRED, ROCS and Align-IT for the benchmarking studies.

ROCS

The X-ray structure of the small molecule co-crystallised in the target protein was used as the query_molecule

The default TanimotoCombo was the scoring function.

Align-IT

For Align-IT the –reference flag was also the corresponding X-ray structure ligand.

DataPype Library Dependencies


```
name: DataPype
channels:
  - anaconda
  - defaults
  - conda-forge
dependencies:
  - _libgcc_mutex=0.1=conda_forge
  - _openmp_mutex=4.5=1_gnu
  - blas=1.0=openblas
  - boost=1.74.0=py310h7c3ba0c_5
  - boost-cpp=1.74.0=h6cacc03_7
  - brotli=1.0.9=he6710b0_2
  - bzip2=1.0.8=h7f98852_4
  - ca-certificates=2020.10.14=0
  - cairo=1.16.0=ha12eb4b_1010
  - certifi=2020.6.20=pyhd3eb1b0_3
  - cycler=0.11.0=pyhd3eb1b0_0
  - expat=2.4.8=h27087fc_0
  - font-ttf-dejavu-sans-mono=2.37=hab24e00_0
  - font-ttf-inconsolata=3.000=h77eed37_0
  - font-ttf-source-code-pro=2.038=h77eed37_0
  - font-ttf-ubuntu=0.83=hab24e00_0
  - fontconfig=2.13.96=h8e229c2_2
  - fonts-conda-ecosystem=1=0
  - fonts-conda-forge=1=0
```

- freetype=2.10.4=h0708190_1
- gettext=0.19.8.1=h73d1719_1008
- giflib=5.2.1=h7b6447c_0
- icu=69.1=h9c3ff4c_0
- jbig=2.1=hdba287a_0
- joblib=0.17.0=py_0
- jpeg=9d=h7f8727e_0
- lcms2=2.12=h3be6417_0
- ld_impl_linux-64=2.36.1=hea4e1c9_2
- lerc=3.0=h295c915_0
- libblas=3.9.0=13_linux64_openblas
- libcblas=3.9.0=13_linux64_openblas
- libdeflate=1.8=h7f8727e_5
- libffi=3.4.2=h7f98852_5
- libgcc-ng=11.2.0=h1d223b6_14
- libgfortran-ng=7.5.0=hae1eefd_17
- libgfortran4=7.5.0=ha8ba4b0_17
- libglib=2.70.2=h174f98d_4
- libgomp=11.2.0=h1d223b6_14
- libiconv=1.16=h516909a_0
- liblapack=3.9.0=13_linux64_openblas
- libnsl=2.0.0=h7f98852_0
- libopenblas=0.3.18=hf726d26_0
- libpng=1.6.37=h21135ba_2
- libstdcxx-ng=11.2.0=he4da1e4_14
- libtiff=4.3.0=h6f004c6_2
- libuuid=2.32.1=h7f98852_1000

- libwebp=1.2.2=h55f646e_0
- libwebp-base=1.2.2=h7f8727e_0
- libxcb=1.13=h7f98852_1004
- libxml2=2.9.12=h885dcf4_1
- libzlib=1.2.11=h166bdaf_1014
- lz4-c=1.9.3=h9c3ff4c_1
- matplotlib-base=3.5.1=py310h23f4a51_0
- munkres=1.1.4=py_0
- ncurses=6.3=h9c3ff4c_0
- openbabel=3.1.1=py310h154a6b4_3
- openssl=3.0.2=h166bdaf_1
- packaging=21.3=pyhd3eb1b0_0
- pcre=8.45=h9c3ff4c_0
- pip=22.0.4=pyhd8ed1ab_0
- pixman=0.40.0=h36c2ea0_0
- pthread-stubs=0.4=h36c2ea0_1001
- python=3.5
- python-dateutil=2.8.2=pyhd3eb1b0_0
- python_abi=3.5
- pytz=2021.3=pyhd3eb1b0_0
- rdkit=2022.03.1=py310h1c297d8_1
- readline=8.1=h46c0cb4_0
- six=1.16.0=pyhd3eb1b0_1
- smina=2020.12.10=h37f9cb6_0
- sqlite=3.37.1=h4ff8645_0
- threadpoolctl=2.1.0=pyh5ca1d4c_0
- tk=8.6.12=h27826a3_0

- tzdata=2022a=h191b570_0
- wheel=0.37.1=pyhd8ed1ab_0
- xorg-kbproto=1.0.7=h7f98852_1002
- xorg-libice=1.0.10=h7f98852_0
- xorg-libsm=1.2.3=hd9c2040_1000
- xorg-libx11=1.7.2=h7f98852_0
- xorg-libxau=1.0.9=h7f98852_0
- xorg-libxdmcp=1.1.3=h7f98852_0
- xorg-libxext=1.3.4=h7f98852_1
- xorg-libxrender=0.9.10=h7f98852_1003
- xorg-renderproto=0.11.1=h7f98852_1002
- xorg-xextproto=7.3.0=h7f98852_1002
- xorg-xproto=7.0.31=h7f98852_1007
- xz=5.2.5=h516909a_1
- zlib=1.2.11=h166bdaf_1014
- zstd=1.5.2=ha95c52a_0
- pip:
  - fonttools==4.31.2
  - greenlet==1.1.1
  - kiwisolver==1.3.1
  - matplotlib==3.5.1
  - numpy==1.22.3
  - pandas==1.4.2
  - pillow==9.0.1
  - pyparsing==3.0.7
  - reportlab==3.5.67
  - rocker==0.1.4

- scikit-learn==1.0.2
- scipy==1.7.3
- setuptools==61.2.0
- sqlalchemy==1.4.32

DataPype Sample Output Report

```
DATAPYPE OVERALL REPORT
------------------------
Overall Time taken for the complete study: 57.70 mins
INPUT DATASET SIZE
------------------
DATASET     Num_of_Actives   Num_of_Decoys    Total_Num_of_Molecules
fabp4 47    2750  2797
mcr   94    5150  5244
mk01  79    4550  4629
LIGAND PREPARATION
------------------
# DATASET = Name of dataset
# W = Number of worker files
# C = No. of Conformers generated (for FRED/Hybrid Docking)
# TIME = Time taken (in minutes)
DATASET     W     C     TIME
fabp4 8     20    23.47
mcr   8     20    29.44
mk01  8     20    36.20
PROTEIN PREPARATION
-------------------
# O = No. of prepared protein output files generated by Spruce
# TIME = Time taken (in minutes)
DATASET     PDB-ID      O     TIME
fabp4 2nnq  1     0.50
mcr   2aa2  1     0.36
mk01  2ojg  1     0.61
MOLECULAR DOCKING
-----------------
# DOCKING = Name of Software used for Docking
# DATASET = Name of dataset
# TIME = Time taken (in minutes)
DOCKING     DATASET     PDB_Chain   TIME
Fred  fabp4 2NNQ_A_T4B_A-293 5.18
Fred  mcr   2AA2_A_AS4_A-201 3.34
Fred  mk01  2OJG_A_19A_A-360 7.30
BENCHMARKING METRICS
--------------------
# ROC-AUC = Area Under the ROC Curve
# BEDROC = BEDROC at alpha 20.0
# EF1% = Enrichment Factor for 1%
#logAUC = logAUC at 0.1%,100%
```

DATASET	PDB_Chain	DOCKING	ROC-AUC	BEDROC	EF1%	logAUC
fabp4	2NNQ_A_T4B_A-293	Fred	0.87	0.49	20.66	0.03
mcr	2AA2_A_AS4_A-201	Fred	0.66	0.27	13.73	0.10
mk01	2OJG_A_19A_A-360	Fred	0.76	0.35	9.98	0.08

SHAPE

```
-----------------
# METHOD = Name of Software used for Shape
# DATASET = Name of dataset
# TIME = Time taken (in minutes)
# ROC-AUC = Area Under the ROC Curve
# BEDROC = BEDROC at alpha 20.0
# EF1% = Enrichment Factor for 1%
#logAUC = logAUC at 0.1%,100%
```

METHOD	DATASET	ROC-AUC	BEDROC	EF1%	logAUC	TIME
ROCS	fabp4	0.81	0.45	33.90	0.06	0.88
ROCS	mcr	0.61	0.24	11.95	0.10	1.02
ROCS	mk01	0.7	0.27	19.64	0.07	1.18

PHARMACOPHORE

```
-----------------
# METHOD = Name of Software used for Shape
# DATASET = Name of dataset
# TIME = Time taken (in minutes)
# ROC-AUC = Area Under the ROC Curve
# BEDROC = BEDROC at alpha 20.0
# EF1% = Enrichment Factor for 1%
#logAUC = logAUC at 0.1%,100%
```

DATASET	METHOD	ROC-AUC	BEDROC	EF1%	logAUC	TIME
fabp4	Align_it	0.68	0.38	31.78	0.08	13.54
mcr	Align_it	0.64	0.17	3.18	0.09	21.52
mk01	Align_it	0.78	0.43	32.91	0.06	20.89
